# L-proline: a highly effective cryoprotectant for mouse oocyte vitrification

**DOI:** 10.1038/srep26326

**Published:** 2016-07-14

**Authors:** Lu Zhang, Xu Xue, Jie Yan, Li-Ying Yan, Xiao-Hu Jin, Xiao-Hui Zhu, Zhi-Zhu He, Jing Liu, Rong Li, Jie Qiao

**Affiliations:** 1 Center for Reproductive Medicine, Department of Obstetrics and Gynecology, Peking University Third Hospital, No. 49 North Hua Yuan Road, Hai Dian District, Beijing 100191, China; 2 Beijing Key Laboratory of Reproductive Endocrinology and Assisted Reproduction, Beijing 100191, China; 3 Beijing Key Lab of Cryo-Biomedical Engineering and Key Lab of Cryogenics, Technical Institute of Physics and Chemistry, Chinese Academy of Sciences, Beijing, China; 4 Key Laboratory of Assisted Reproduction, Ministry of Education, Beijing 100191, China; 5 Department of Biomedical Engineering, School of Medicine, Tsinghua University, Beijing, China

## Abstract

Recent studies have shown that L-proline is a natural osmoprotectant and an antioxidant to protect cells from injuries such as that caused by freezing and thawing in many species including plant, ram sperm and human endothelial cells. Nevertheless, this nontoxic cryoprotectant has not yet been applied to mammalian oocyte vitrification. In this study we evaluated the efficiency and safety of the new cryoprotectant in oocyte vitrification. The results indicated that L-proline improves the survival rate of vitrified oocytes, protects mitochondrial functions and could be applied as a new cryoprotectant in mouse oocyte vitrification.

Since the first baby was born after oocyte vitrification in 1999^1^, approximately 4,000 live births have been obtained by vitrification. This technique provides an effective option to preserve reproductivity for women with cancer, infertile women, or even fertile women at risk of age-induced fertility decline. Vitrification is characterized by an ultra-rapid cooling time and a glass-like formation to avoid forming ice crystals by high cryoprotectant concentration^2^. The standard vitrification solution consists of 15% (v/v) ethylene glycol (EG), 15% (v/v) dimethy sulphoxide (DMSO), 0.5 mol/L sucrose medium, 20% fetal bovine serum (FBS) and phosphate-buffered saline (PBS)^3^. Although this protocol is currently widely used for routine oocyte cryopreservation and was recommended by American Society of Reproductive Medicine (ASRM) in 2012, the clinical outcome could be further improved because of lower pregnancy and live birth rates resulting from decreased survival rates and poor embryo development. This poor clinical outcome is closely related to osmotic stress, oxidative stress and high concentrations of cryoprotective agents (CPA) such as DMSO that can injure oocyte during freezing and thawing^4^. Although vitrification imparts less stress to the cells, trauma to the human oocytes cannot be avoided. During oocyte dehydration-rehydration cycles, cellular alterations associated with osmotic stress may damage mitochondrial metabolism^5^. Even EG, a less toxic CPA, showed cytotoxic effects on cell viability and development in a dose-dependent manner. As a consequence, it would be critical to minimize the cryogenic injury and the toxicity of CPAs without decreasing the efficiency of vitrification solution.

L-Proline, a natural amino acid, is an ideal candidate for natural cryoprotection since it has a very high solubility, a neutral pH, exerts a high osmotic pressure, is nontoxic at high concentrations, and is classified as an osmoprotectant and an antioxidant. L-Proline was initially found accumulating in plants in response to environmental stress including freezing temperatures. L-Proline protectes yeast and fly larva against the freeze stress, and the freeze tolerance is enhanced with the elevated levels of proline^6^. Because of its role in freeze tolerance *in vivo*, L-Proline was introduced into the *in vitro* cryopreservation in many species such as plant, yeast, fly, ram, and human^7^,^8^,^9^. For example, ram sperm, human mesenchymal stem cells and human endothelial cells were successfully frozen under a low level of L-proline^7^,^8^,^9^. Nevertheless, this nontoxic cryoprotectant has not yet been applied to mammalian oocyte vitrification.

We aim in this study to evaluate the vitrification efficiency of L-Proline combined with DMSO and EG in mouse oocytes.

## Results

### L-proline improves the survival rate of oocytes after vitrification and thawing

At the beginning, we selected 2 mol/L as the experimental concentration of L-proline, which was optimized for osmolarity. To assess the efficiency of L-proline, we compared two groups: the proline-free group (control) and the proline group 1 (add 2 mol/L L-proline to the control group). To further reduce the toxicity of vitrification solution, we investigated another group: the proline group 2 (consisting of 2 mol/L L-proline and low levels of DMSO and EG). In the preliminary experiment, the survival rate of thawed oocytes vitrified in 2 mol/L L-proline with 7.5% DMSO and 10% EG was significantly higher than that of 2 mol/L L-proline with other lower concentrations of DMSO and EG, when the concentrations of DMSO and EG were decreased gradually. That is why we selected 2 mol/L L-proline with 7.5% DMSO and 10% EG as the experimental proline group 2.

The survival rates of vitrified-warming oocytes in the proline-containing groups (544 oocytes in proline group 1 and 553 oocytes in proline group 2) were significantly higher than that of the proline-free group (630 oocytes) (94.7%, 93.7% vs. 88.4%, *P* < 0.05) (Table 1).

### L-proline maintains the normal spindle configuration and protects oocytes from mitochondrial damage

**Figure 1:**
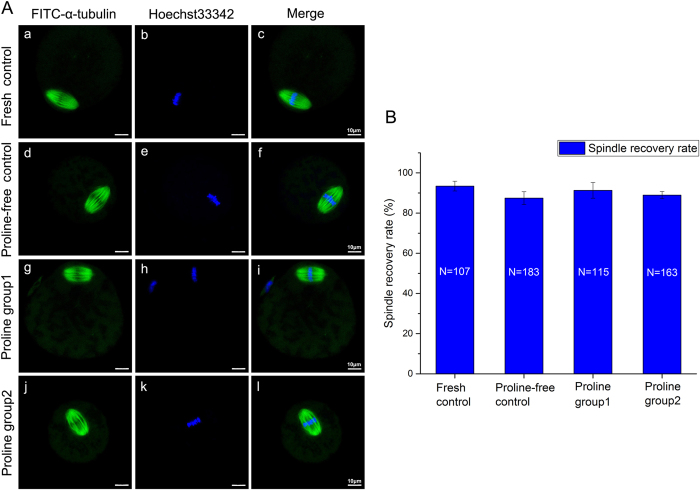
Analysis of spindle configuration of oocytes in fresh control (a–c) and vitrified-thawing oocytes in proline-free control (d–f), proline group 1 (g–i) and proline group 2 (j–l) 2 hours after thawing. Panel **A**: The immunofluorescence photographs of meiotic spindle morphology and chromosome alignment in these groups. Green color: FITC-α-tubulin; Blue color: Hoechst33342-chromosome. Panel **B**: Means ± SE of the spindle recovery rate in these groups. N: The number of oocytes in each group.

After 2 hours of incubation at 37 °C, the majority of fresh oocytes and thawed oocytes in the proline-free group and the proline-containing groups maintained normal meiotic spindle morphology and chromosome alignment (93.46%, 87.43%, 91.30%, 88.96%, respectively, *P* > 0.05) (Fig. 1A,B).

**Figure 2:**
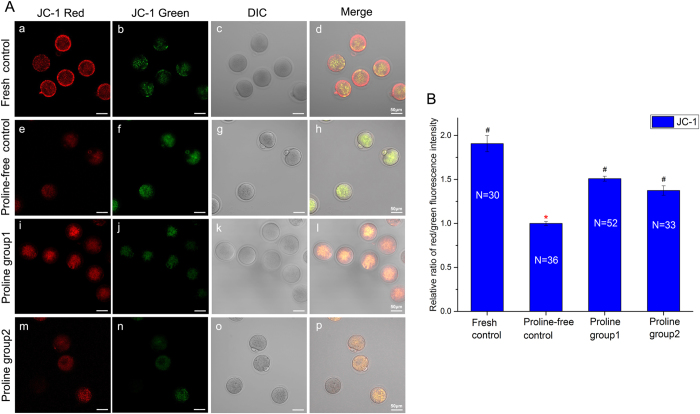
MMP assessment of oocytes in fresh control (a–d) and vitrified-thawing oocytes in proline-free control (e–h), proline group 1 (i–l) and proline group 2 (m–p). Panel **A**: The fluorescence photographs of MMP detected by JC-1 in these groups. Red color: Hyperpolarized mitochondria (a,e,i,m); Green color: Lower MMP (b,f,j,n). Panel **B**: Comparison of the relative ratios of red/green fluorescence intensities stained by JC-1 in these groups. *#Values with different superscripts are significantly different (Mean ± SE, P < 0.05). No differences between values with the same superscript. N: The number of oocytes in each group.

Furthermore, we detected the Inner Mitochondrial Membrane Potential (MMP) of the vitrified-thawing oocytes and the unvitrified oocytes by JC-1 staining. Lower ratio of red/green fluorescence intensity represented lower MMP and more serious mitochondrial damage^10^. In Fig. 2, the ratios of red/green optical density captured from the proline-containing groups were much higher than that of the proline-free control group (*P* < 0.05), but similar to the fresh group (*P* > 0.05) (Fig. 2A,B). It indicated that the MMP of non-proline-vitrified oocytes was significantly lower than that of unvitrified oocytes. L-proline protected the MMP in the oocytes against vitrified mitochondrial injury.

**Figure 3:**
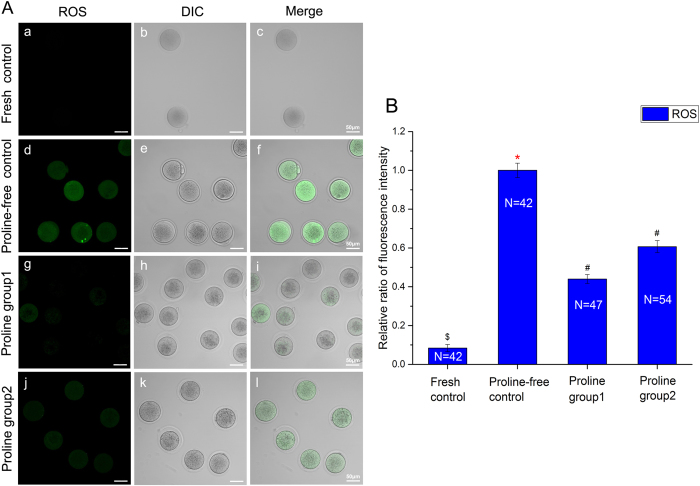
ROS assessment of oocytes in fresh control (a–c) and vitrified-thawing oocytes in proline-free control (d–f), proline group 1 (g–i) and proline group 2 (j–l). Panel **A**: The fluorescence photographs of ROS detected by DCFH-DA in these groups. Green color: ROS (a,d,g,j). Panel **B**: Comparison of the relative ratios of ROS fluorescence intensities in these groups. ^*#$^Values with different superscripts are significantly different (Mean ± SE, P < 0.05). No differences between values with the same superscript. N: The number of oocytes in each group.

Intracellular Reactive Oxygen Species (ROS) is a sensitive index to evaluate the oxidative stress in oocytes. The relative ratio of DCFH-DA fluorescence intensities of the vitrified groups were much higher than that of the fresh oocytes (*P* < 0.05, Fig. 3A,B). But in contrast to the proline-free group, it was much lower in the proline-containing groups (*P* < 0.05, Fig. 3A,B).

**Figure 4:**
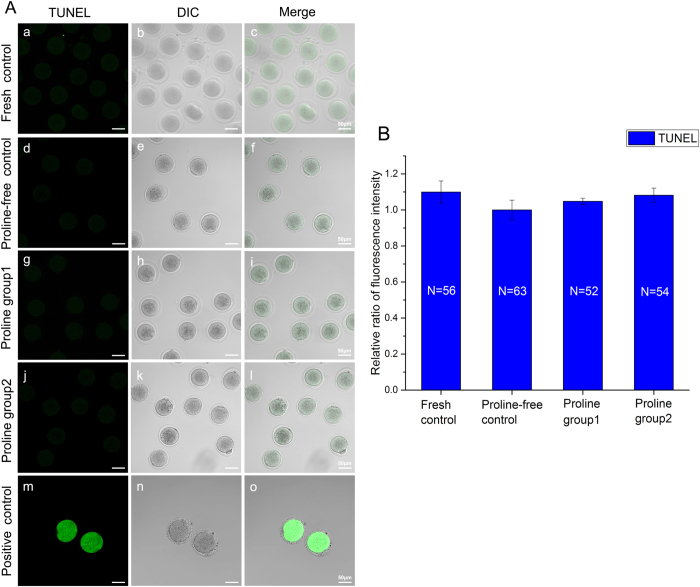
Apoptosis assessment of oocytes in fresh control (a–c) and vitrified-thawing oocytes in proline-free control (d–f), proline group 1 (g–i) and proline group 2 (j–l). Panel **A**: The fluorescence photographs of apoptosis detected by TUNEL assay in these groups. Green color: TUNEL staining positive (a,d,g,j,m). The bottom panel (m–o) is the positive control. Panel **B**: Comparison of the relative ratios of TUNEL fluorescence intensities in these groups.

For apoptosis analysis, we detected by TUNEL assay. As presented in Fig. 4, the relative ratio of TUNEL fluorescence intensities in the proline-vitrified oocytes were similar to that of the non-proline-vitrified oocytes (*P* > 0.05, Fig. 4A,B). There was no significant difference between the vitrified group and the unvitrified group (*P* > 0.05, Fig. 4A,B).

### L-proline does not affect the potential of early embryonic development *in vitro* and *in vivo*

We observed the embryonic development derived from the vitrified-thawing oocytes. After ICSI, the two pronuclei rates of the proline-containing groups were equivalent to that of the proline-free group and the fresh group (*P* > 0.05, Table 1). The ICSI rate, two-cell formation rate and blastocyst rate of the proline-containing groups were similar tothe proline-free group, but less than that of the fresh group (Table 1).

The implantation rate and the live birth rate of the proline-containing groups were consistent with that of the proline-free group, but lower than that of the fresh group (Table 1). There was no congenital malformation among the live births of the proline-containing groups, the proline-free group and the fresh group. The birth weight, body length and the sex ratio of fetuses have no significant differences between the vitrified groups and the fresh group (Table 1).

## Discussion

First of all, it is the first time to apply L-proline in mouse oocyte vitrification. In our study, L-proline makes higher survival rate and better mitochondrial function in vitrified-thawing oocytes of proline group 1 in contrast with control. One of the explanations is that L-proline is classified as an osmoprotectant and an antioxidant. As been reported, human oocytes injury caused by osmotic stress may be associated with the abnormal mitochondrial membrane structures during dehydration-rehydration cycles^11^,^12^. In addition, the process of freezing and thawing increases production of ROS which may induce release of cytochrome C and other apoptogenic factors from cell mitochondria, and it could eventually lead to programmed cell death^12^. However, L-proline can reduce the excessive ROS to prevent oocytes from oxidative stress damage. An ROS-scavenging mechanism that is important to proline stress protection is the facile reaction of proline with singlet oxygen^13^. Pyrrolidine, which forms the 5-membered ring of proline, has a low ionization potential that effectively quenches singlet oxygen most likely through a charge transfer mechanism in which molecular oxygen returns to the ground triplet state^14^. In plant cells, accumulate proline is considered to be a positive index for osmotic stress resistance^15^, and a ROS scavenger to reduce the potential for oxidative damage.

Secondly, L-proline is safe and nontoxic and could be used in oocyte vitrifcation. It improves the mitochondrial function (Figs 2, 3, 4) and has no adverse effects on the spindle configuration (Fig. 1) and the *in vitro* and *in vivo* embryonic development (Table 1). Previous reports showed that mitochondria from freezing-thawed human oocytes possess decreased matrix electron density or have ruptures of the outer and inner membranes caused by cryopreservation^16^, which can lead to the malformation during embryonic development^17^. Freezing procedures can also directly result in DNA fragmentation and cell degeneration via apoptosis in MII bovine ocytes^18^. Therefore, we choose the parameters of spindle recovery, mitochondrial function and embryonic development for safety assessment.

Basically, L-proline is natural and nontoxic at high concentrations. And excessive L-proline inside the cell could be catabolized in mitochondria by degradation pathways^19^. The first step is the oxidation of proline to ∆1-pyrroline-carboxylate (P5C)^19^. Then the P5C generated is converted to glutamate^20^. Unnecessary L-proline is metabolized in this physiologic way. Furthermore, due to its action as a singlet oxygen quencher, proline may help reduce oxidative damage to vital cellular macromolecules and consequently stabilize proteins^21^, DNA^22^ and membranes^23^ to maintain normal meiotic spindle morphology, avoid apoptosis and protect mitochondrial membrane. Consistently, data from *in vitro* and *in vivo* embryonic development (Table 1), demonstrate that L-proline has no adverse effects on embryo development after vitrification and thawing. Although no obvious differences were observed in post-fertilization development between the proline-containing and proline-free group on the surface, there might be some phenotypic and epigenetic changes in the offspring during later long-term development. Indeed, L-proline improved the MMP and decreased the ROS in the mouse oocytes after vitrification and thawing. In the later and long-term development study, we prepare to investigate the neurobehavioral manifestation, epigenetic modification and reproductive alteration of those offspring derived from proline-vitrified oocyte.

Thirdly, we optimize a new vitrification solution with L-proline and lower DMSO and EG (proline group 2). It has already been confirmed that L-proline is a nontoxic cryoprotectant and could be applied in mouse oocyte vitrification earlier (proline group 1). In order to reduce the total toxicity of vitrification solution, we combine L-proline with low concentration of DMSO and EG. It was proved that high concentrations of DMSO and EG were toxic to oocyte viability and development during freezing and thawing^4^. In addition, both of external and internal releases of calcium indicated that DMSO may directly impair the abilities of mitochondria and endoplasmic reticulum^24^. It is critical to minimize the concentrations and the toxicities of DMSO and EG without decreasing the efficiency of vitrification solution. As is shown in Table 1, the survival rate of vitrified oocyte in proline group 2 with lower DMSO and EG is much higher than that of control (proline-free group). Interestingly, lower levels of DMSO and EG are used in proline-containing group (proline group 2), less harmful damage to mitochondria is found (Figs 2, 3, 4). More than that, there are no adverse effects on spindle configuration and embryonic development in proline group 2 (Fig. 1, Table 1). It indicates that L-proline combining with low concentrations of DMSO and EG could be a new vitrification solution appropriately for mouse oocyte vitrification.

In conclusion, L-proline can be applied as a new cryoprotectant initially in mouse oocyte vitrification. And a new vitrification solution consisting of 2 mol/L L-proline, 10% EG, 7.5% DMSO and 0.5 mol/L sucrose medium with hypotoxicity is more suitable for mouse oocyte vitrification. More research is required to determine the cryoprotective effects of L-proline in other mammalian species like human oocytes and to determine the phenotypic changes of the offsprings from the vitrified mammalian oocytes.

## Methods

### Oocytes collection

Female B6D2F1 mice (Vital River Laboratory Animal Technology Co. Ltd., Beijing, China) were maintained according to the Chinese National Standard (GB14925–2001). Denuded metaphase II (MII) oocytes were obtained from super-ovulated mice. All procedures involving mice were operated under strict criteria on the basis of the Guide for Care and Use of Laboratory Animals of Peking University, and the animal experiments were approved by the Institutional Animal Welfare and Ethics Committee of Peking University (No. LA2012-12).

### Vitrification solution

MII oocytes were divided into four groups: fresh group (without vitrification), proline-free control group (vitrified-thawed in solution: 15% EG (Sigma, cat.no. 293237, USA), 15% DMSO (Sigma, cat.no.D2650, USA), 0.5 mol/L sucrose medium (Sigma, cat.no.V900116, USA) and 20% FBS (Thermo Fisher, cat.no. 10099-141, Australia) in PBS (Thermo Fisher, cat.no. 14190-250, USA)), proline group 1 (vitrified-thawed in solution: 2 mol/L L-proline (Sigma, cat.no.P5607, USA), 15% EG, 15% DMSO, 0.5 mol/L sucrose medium and 20% FBS in PBS), proline group 2 (vitrified-thawed in solution: 2 mol/L L-proline, 10% EG, 7.5% DMSO, 0.5 mol/L sucrose medium and 20% FBS in PBS).

### Oocyte vitrification and thawing

Mature oocytes were vitrified by a standard procedure. Briefly, oocytes were equilibrated in a 7.5% EG plus 7.5% DMSO (v/v) solution for 5 minutes at room temperature. Then oocytes were exposed to the vitrification solutions respectively for less than 1 minute at 25 °C and immediately loaded on sterile iVitri straw (Reprobiotech Corp., cat.no.RBC-S-008, USA). And the iVitri straws were plunged directly into liquid nitrogen and stored at −190 °C for at least 1 month.

Thawing was carried out by five steps using sucrose solutions in PBS containing 20% FBS at room temperature. First the oocytes were expelled from the straws into 1.0 mol/L sucrose for 3 minutes, then successively transferred into 0.5 mol/L, 0.25 mol/L and 0 mol/L sucrose medium each for 3 minutes, and finally the thawed oocytes were cultured in human tubal fluid (HTF) (LifeGlobal, cat.no.LGGF-100, Belgium) medium at 37 °C in 5% CO_2_ of a humidified air, and assessed for survival rate. After incubation for 2 hours, the oocytes were ready for further research. The oocyte survival was characterized by the morphological appearance of membrane integrity and discoloration of the ooplasm^25^. And the survival rate of the thawed oocytes was assessed 2 hours after incubation^25^. The survival oocytes were used for spindle analysis, mitochondrial analysis and embryo development assessment.

### Immunofluorescence

For spindle analysis, vitrified-thawing oocytes were detected by α-tubulin-FITC antibody (diluted 1:100 in PBS; Sigma; cat.no.F2168). Images were captured by a confocal laser scanning microscope (LSM710 Carl Zeiss, Oberkochen, Germany).

### Inner mitochondrial membrane potential (MMP) and Reactive Oxygen Species (ROS) staining

To evaluate MMP and ROS, fresh and the post-thaw oocytes were detected by JC-1 fluorochrome (Invitrogen, cat.no.M34152, Carlsbad, CA) or DCFH-DA (Nanjing Jiancheng Bioengineering Institute, cat.no.E004, Nanjing, China).

### TUNEL Apoptosis Assay

For Terminal Deoxynucleotideyl Transferase-mediated dUTP Nick-End Labeling (TUNEL), fresh and warmed oocytes were applied by TUNEL kit (Beyotime Biotechnology, cat.no.C1086, Shanghai, China).

### Intracytoplasmic sperm injection (ICSI)

Before ICSI, the sperm suspensions, retrieved from the cauda epididymis of 10-week-old ICR male mice (Vital River Laboratory Animal Technology Co. Ltd., Beijing, China), were capacitated first. Fresh and vitrified-thawing oocytes were microinjected using a Piezo drill (PMM Controller, Prime Tech, Ibaraki, Japan) as described^26^. The ICSI rate referred to the percentage of survival oocytes after ICSI. Then fertilized oocytes with a second polar body or two pronuclei (2PN) were removed and cultured in GM media (lifeGlobal, cat.no.LGGG-50, Belgium) to two-cell stage under 5% CO_2_ in humidified air at 37 °C. Part of two-cell embryos were cultured *in vitro* to blastocyst stage and the rests were used for embryo transfer for *in vivo* development.

### Embryo transfer

Pseudopregnant ICR female mice were mated with vasectomized ICR male mice. Then two-cell embryos were transferred into the oviduct of pseudopregnant ICR female 0.5 day post-coitus. Some mice delivered naturally and the others delivered by cesarean section to analyze embryo implantation on day 19.5. Each fetus was examined for macroscopic malformations, and measured by weight and body length at birth.

### Assessment of implantation and post-implantation development

Some of the pregnant female mice were sacrificed by cervical dislocation at 19.5 day post-coitus. The uterine contents were examined. The number of live births, stillborns and early and late resorptions in each uterine horn was recorded. The implantation rate was calculated as: total number of live births, stillborns and resorptions/number of embryos transferred. The live birth rate represented the total number of live birth by vaginal delivery and cesarean delivery/the total number of two-cell embryos transferred.

### *In vitro* embryo culture

Fertilized zygotes by ICSI were cultured in GM media until blastocyst stage under 5% CO_2_ in humidified air at 37 °C. Two pronuclei rate was recorded 12 hours after culture, two-cell rate was recorded on day 1 of culture and blastocyst rate was recorded on day 4 of culture.

### Statistical analysis

Differences among groups were analyzed with Chi square test for categorical data and ANOVA for numeric data using SPSS 20.0 software. Results were expressed as Mean ± SE. *P* < 0.05 was considered statistically significant. For all results, the examples shown are representative of at least three replications.

## Additional Information

**How to cite this article**: Zhang, L. *et al.* L-proline: a highly effective cryoprotectant for mouse oocyte vitrification. *Sci. Rep.* **6**, 26326; doi: 10.1038/srep26326 (2016).

## Figures and Tables

**Table 1:** The development of ICSI oocytes after vitrification and thawing in mouse.

	Fresh group	Proline-free control	Proline group 1	Proline group 2
Survival rate %(n/n)	–	88.4 (557/630)^a^	94.7 (515/544)^b^	93.7 (518/553)^b^
ICSI rate %(n/n)	88.2 (157/178)^a^	79.0 (184/233)^b^	80.1 (173/216)^b^	81.8 (175/214)^b^
Two pronuclei rate (per oocyte) %(n/n)	94.9 (149/157)^a^	92.4 (170/184)^a^	93.6 (162/173)^a^	93.7 (164/175)^a^
Two-cell rate (per oocyte) %(n/n)	90.4 (142/157)^a^	76.1 (140/184)^b^	80.3 (139/173)^b^	79.4 (139/175)^b^
Blastocyst rate (per oocyte) %(n/n)	75.8 (72/95)^a^	51.4 (54/105)^b^	54.1 (53/98)^b^	52.4 (54/103)^b^
Implantation rate (per two-cell transferred) %(n/n)	80 (32/40)^a^	52.5 (21/40)^b^	57.5 (23/40)^b^	55 (22/40)^b^
Live birth rate (per two-cell transferred) %(n/n)	60.7 (34/56)^a^	38.3 (23/60)^b^	40 (24/60)^b^	38.6 (22/57)^b^
Healthy live birth rate %(n/n)	100 (34/34)^a^	100 (23/23)^a^	100 (24/24)^a^	100 (22/22)^a^
Birth weight (1 day old) (g)	1.70 ± 0.02^a^	1.77 ± 0.02^a^	1.74 ± 0.02^a^	1.76 ± 0.01^a^
Birth body length (1 day old) (cm)	2.85 ± 0.02^a^	2.91 ± 0.02^a^	2.85 ± 0.02^a^	2.90 ± 0.02^a^
Sex ratio (female/male)	19/15^a^	15/8^a^	9/15^a^	10/12^a^

Note: ^ab^Values with different superscripts in the same line are significantly different (P < 0.05). The data is shown as Means ± SE.
